# Interpretation guidance for MHRA regulatory considerations for phage therapeutic products

**DOI:** 10.1099/mic.0.001613

**Published:** 2025-11-13

**Authors:** Carmen Coxon, Elizabeth Bell, Evelien Adriaenssens, Jason Clark, Joe Edwards, Tas Gohir, Francesca Hodges, Josh Jones, Cath Rees, Annette Sansom, Darren Smith, Mark Sutton, Clare Trippett, Dann Turner

**Affiliations:** 1 MHRA Science Campus, Blanche Lane, South Mimms, Potters Bar, Hertfordshire, EN6 EQG, UK; 2 National Institute for Health and Care Excellence, Manchester, UK; 3 Innovate UK Phage Innovation Network Advisory Group, Unit 218, Business Design Centre, 52 Upper Street, Islington, London N1 0QH, UK; 4 Bacteriophages and Viromics, Quadram Institute, Rosalind Franklin Road, Norwich Research Park, Norwich NR4 7UQ, UK; 5 NexaBiome, West of Scotland Science Park, Block 2 Kelvin Campus, 2317 Maryhill Road, Glasgow G20 0SP, UK; 6 Aparon, Hill Farm Middleton Lane, Middleton, Tamworth, B78 2BN, UK; 7 Guy’s and St Thomas' NHS Foundation Trust, The Grain House, 46 Loman Street, London SE1 0EH, UK; 8 Innovate UK Business Connect, Unit 218, Business Design Centre 52 Upper Street, Islington, London N1 0QH, UK; 9 Antimicrobial Discovery Development and Diagnostics (AD3), UK Health Security Agency, Manor Farm Road, Porton Down, Salisbury, Wiltshire SP4 0JG, UK; 10 School of Biosciences, University of Nottingham, Nottingham, UK; 11 Campden BRI, Station Road, Chipping Campden, Gloucestershire GL55 6LD, UK; 12 Applied Sciences, Northumbria University, Newcastle upon Tyne, NE1 8ST, UK; 13 Institute of Pharmaceutical Sciences, King’s College London, London, UK; 14 Centre for Process Innovation, 1 Union Square, Central Park, Darlington DL1 1GL, UK; 15 University of the West of England, Bristol, Coldharbour Lane, BS16 1QY, UK; 16 School of Applied Sciences, College of Health, Science and Society, University of the West of England, Bristol, Coldharbour Lane, BS16 1QY, UK

**Keywords:** critical quality attribute, personalized medicine, phage therapeutic products, regulation

## Abstract

On 4 June 2025, the MHRA published ‘Regulatory considerations for therapeutic use of bacteriophages in the UK’. This was in response to recommendations made by the House of Commons Science, Innovation and Technology Committee Inquiry into the ‘The Antimicrobial Potential of Bacteriophages’. The MHRA Regulatory Considerations for phage therapeutic products (PTPs) outlines the relevant regulatory route and requirements to use PTPs as licensed or unlicensed medicines. While this guidance provides the necessary information, it is recognized that regulatory information can be inaccessible to academic and small- to medium-sized enterprise developers who are often unfamiliar with the language, terminology and location of such information. The MHRA, in consultation with the Innovate UK Phage Innovation Network, has therefore developed this interpretation to help PTP developers understand what the guidance is saying, and what evidence is required for regulatory assessment of a marketing authorization application. Examples have been included throughout to provide context and as an aid to understanding.

## Introduction

At its best, regulation enables innovation, maximizing benefit but minimizing harm to the intended user by identifying risks and ensuring they are understood and mitigated where possible. At its worst, regulation impedes innovation. Sometimes this is because regulation lags behind innovation, but sometimes it is because information is inaccessible, often leading to confusion and the perpetuation of misinformation.

Innovators can also be frustrated by the time and resources needed to meet regulatory requirements as they want to get products to where they are needed quickly. Regulators also want this, but it is their legal obligation to ensure that products meet quality, safety and efficacy requirements. To help innovators anticipate and meet evidence requirements for regulatory evaluation, regulatory guidelines are produced to help innovators understand what evidence is needed to enable regulatory evaluation of their products. Guidelines are there to provide top-level guidance for as many products as possible, with some lower-level guidance or annexes for products for which the high-level information isn’t detailed enough or appropriate. However, too much detail around expectations can become restrictive as it can quickly become outdated. Developing regulatory guidance that provides enough detail but not too much is a difficult balance to strike.

For novel products that don’t align with any existing regulatory frameworks, it can be difficult to understand what evidence is needed for regulatory evaluation. It is important that, in such cases, developers and manufacturers speak to regulators to inform them about their product and the processes and analytical techniques used to manufacture and validate them. Innovators should be working **with** regulators; the competent authority evaluates the evidence, but it is up to the product developer/manufacturer to make sure that the right evidence is provided for the competent authority to evaluate. Bacteriophages (phages) are a particularly interesting case because they are not a new technology, but they are new to modern regulatory frameworks. This publication is intended to provide information about both regulation as a subject, to enable the reader to interpret regulatory requirements and to understand how phages are viewed from a regulatory perspective. The hope is that this paper helps those developing phage therapeutic products understand regulatory thinking, apply it to their product and communicate effectively with regulatory bodies.

## Regulatory harmonization

The MHRA ‘Regulatory considerations for therapeutic use of bacteriophages in the UK’ [1] outlines all the regulatory pathways that exist for bringing phage products for human therapy to the UK market. This guidance aligns with many other national regulatory authorities (NRAs) who observe harmonized international guidelines developed by the International Council for Harmonisation of Technical Requirements for Pharmaceuticals for Human Use (ICH). ICH regulatory guidelines provide a consensus on evidence requirements for quality, safety and efficacy (veterinary guidance is developed and published by the VICH). At times, we use phage-based examples as an aid to explain concepts – these might be suitable at the time of writing, but as the field evolves, they may cease to be appropriate; developers are the experts on their product(s) and should decide how to demonstrate quality, safety and efficacy.

## Regulatory frameworks, phage engineering and environmental considerations

### Regulatory frameworks applicable to the use of PTPs in the UK health system

Medicine regulation is not the only regulatory framework that applies to medicines. Medicine regulators provide market access via a licence, but procurement and healthcare system implementation run under separate governance systems, and these are country-specific. Furthermore, environmental regulation may also apply. With regard to product adoption and cost evaluation, the UK has three health technology assessment (HTA) bodies who carry out cost-benefit analysis on medicinal products – the evidence needed for cost evaluation may not be the same as that required by the MHRA to grant market access. In the UK, there are three HTA bodies – the Scottish Medicines Consortium (SMC), the National Institute for Health and Care Excellence (NICE) and the All Wales Medicines Strategy Group (AWMSG). Product developers are encouraged to engage with HTAs early, and we have signposted the relevant information for UK HTAs below and highlighted why considering HTA requirements early on might be important.

### Regulatory frameworks for natural and engineered phages

Many regulators have categorized phages as biologics, with genetic modifications tying in other areas of regulation. The Food and Drug Administration (FDA) definition of a human gene therapy product excludes engineered phage products because the genetic modification does **not target the human recipient*****,*** but EU definitions do not make this distinction, so if a genetic change is linked to mechanism of action (MoA), it meets the definition of a gene therapy medicinal product and advanced therapeutic medicinal product (ATMP). The EMA/CAT/600280/2010 Committee for Advanced Therapies (CAT) reflection paper on classification of ATMPs states that a gene therapy must (a) contain recombinant nucleic acid(s) that (b) should be directly involved in the mechanism of therapeutic action of the product. Therefore, in the UK and EU:

1. **Natural phages** are classed as biological medicines.
2. **Engineered phages** containing genetic modifications **not** directly linked to MoA, i.e. to improve quality and/or safety, e.g. reduced immunogenicity, increased yield or stability, would also be biologics as above, but they would have genetically modified micro-organism (GMM) status and, in the UK, require an assessment from the Department for Environment, Food and Rural Affairs (Defra) for deliberate release, and/or the Health and Safety Executive (HSE) for contained use.
3. **Engineered phages carrying genetic modifications linked to MoA** e.g. increased specificity/efficacy towards a target, or new/exogenous genes (payloads) that kill target bacteria, are classed as gene therapy medicinal products in the UK and EU. Again, these are GMMs, and market authorization (MA) can only be granted if there has been an environmental assessment signed off by Defra and/or HSE.

Guidance documents, such as EMA’s Pre-authorisation guidance, contain information on environmental risk assessments (ERAs) of genetically modified medicinal products for human use. There is available regulatory guidance on the use of genetically modified organisms (GMOs in the UK [2,4], as well as examples of approved applications for the controlled release of GMMs, including pharmaceutical GMMs [2]. EMA guidance for Environmental Risk Assessment pertaining to GTMPs is also available [5].

Where a product does not sit within an existing framework, or might be better aligned to one framework rather than another, it is highly recommended that an inquiry is made to the intended regulator at the earliest opportunity. In the context of GMM phage products, a summary of the product and details of the genetic modification and how it relates to the MoA is required to allow assessors to advise you on the most suitable regulatory framework and evidence needed to evaluate quality, safety and efficacy. The EMA has a collection of regulatory guidance pertaining to gene therapy medicinal products [6] and the EMA's quick guide and checklist for ATMPs: Clinical Development [7].

### Regulatory categories of phage therapeutic products in the UK

Phages isolated or manufactured with the intention of treating a medical condition are classified as biological medicinal products or ATMPs and are consequently subject to the Human Medicines Regulations 2012 (HMRs, as amended). ATMPs are also subject to both the HMRs and separate legislative requirements. The MHRA Regulatory Considerations for Therapeutic Use of Bacteriophages in the UK [1] outlines the definitions and legal status of licensed and unlicensed medicines (including ‘specials’) and section 10 exemptions (see MHRA guidance for definition).

## Introducing quality, safety, efficacy and intended use

Quality, safety, efficacy and intended use of medicinal products should be considered from product inception and throughout its lifecycle. They are the cornerstones of the ICH Common Technical Document (CTD) [8], which many regulators observe. The ICH provides comprehensive guidance for each CTD module, including for biologics and cell therapy medicinal products. Quality, safety and efficacy are interconnected, and this interconnectivity builds and expands continually throughout development. For example, safety and efficacy data at later stages of development inform what constitutes key product quality attributes, which then continue to be defined as clinical studies and manufacturing processes develop.

### Quality

In the regulatory context of a medicinal product, the ‘quality’ of a product relates to the biochemical and biophysical characteristics of the product, especially those that relate to safety and efficacy.

Many phage therapy products will be classified as biologics. Many biological medicinal products have key post-translational modifications (PTMs) that affect their activity such as glycosylation [9,10] or tyrosine sulphation [11,12], some of which are linked directly to safety and/or efficacy. Recently, phage PTMs have been linked to adaptability and host immune cell evasion [13]. Critically, changes in manufacturing processes can affect these ‘quality attributes’; defining and monitoring product quality is important throughout the product lifecycle but it is the primary focus when starting product development. Comprehensive characterization of a product helps to monitor and detect changes in quality, identity, purity or activity over time. Batch-to-batch variation in biological medicines is well known and accounted for in regulatory guidance.

ICH Q8(2) Pharmaceutical Development provides an overview of how quality, risk and product development are linked. Understanding a product’s physicochemical properties (its ‘quality’), and biological activity/MoA, helps identify potential risks to those attributes and mitigate them through, e.g. modifications to the drug substance (DS), formulation, storage conditions or manufacturing processes.

There are, at the time of writing, 14 ICH quality guidance documents [14], some of which are further broken down into subcategories. Please note that quality also applies to data integrity; good laboratory practice (GLP) should be adhered to, and the MHRA has information applicable to the UK [15]. Further, ‘any test facility which conducts, or intends to conduct, regulatory studies, – including pharmaceuticals, must comply with good laboratory practice GLP regulations’.

On the 16th October 2025, the EMA published ‘Guideline on quality aspects of phage therapy medicinal products’ for public consultation [16]. This document, which at the time of writing is in draft and subject to change, provides guidance on regulatory expectations for PTM quality and the associated evidence that must be provided for review. In section 3 of this draft document, it is stated that the guidance should be read in conjunction with ICH guidelines applicable to PTMs as well as Ph. Eur. requirements; the MHRA ‘Regulatory considerations for therapeutic use of bacteriophages in the UK’ provides clarity on the former (ICH guidelines applicable to PTMs).

### Safety

Once your material is well characterized - its ‘quality’ is well understood and it can be produced as consistently and reproducibly as possible - it can be investigated for safety in preclinical programmes. This will provide an understanding of how the material will behave in a biological context. Regulators acknowledge that animal models are not always fit for purpose [17], and, furthermore, regulators are obligated to support the 3Rs (Replacement, Reduction and Refinement) in research and minimize the use of animals; animal studies should only be used when they add value. Be rational in choosing approaches and methodologies and think about what a regulator would want to see to be confident that everything has been done to understand the drug substance/drug product (DP) before moving into human studies. Quality is an important consideration here too – the equipment, materials and assays should be appropriate, reproducible and reliable, and the inclusion of reference reagents and quality controls (QCs) will be important to demonstrate consistency and be confident in the data. For regulatory expectations around the use of reference materials to validate methodologies and product quality measurement, see below.

### Efficacy

Once product **quality** and **safety** attributes are understood and QC parameters are defined, the focus can shift to efficacy. Appropriate assays to measure potency are key, but it is essential that quality is applied to record keeping also to ensure full traceability around data, documentation and processes, as well as materials and reagents used for experimental work.

### Intended use

Product development strategies must be designed with the end user in mind – the active pharmaceutical product treats the disease, but the **drug product** (DP must be suited to the geography, culture, climate, local infrastructure, economy and genetics of the population it is intended for. These factors will influence design and manufacture and, therefore, quality, safety and efficacy. It is also wise to investigate specific NRA requirements in the country of intended registration; while efforts are made to harmonize regulatory requirements, submissions and processes, this is not always the case (or possible), and it is essential that specific requirements are identified and accounted for at the earliest opportunity.

### Reference reagents

Reference materials should be used to validate methods and provide confidence to regulators about their reproducibility and suitability, where possible. ICH chapter 6QB [18] acknowledges that such materials are not always available for new types of biologics, stating, ‘At the time of [MA] submission, the manufacturer should have established an appropriately characterised in-house primary reference material, prepared from lot(s) representative of production and clinical materials. In-house working reference material(s) used in the testing of production lots should be calibrated against this primary reference material. Where an **international or national standard** is available and appropriate, reference materials should be calibrated against it’. The importance of reference materials is also stated in the EMA Guideline on quality aspects of phage therapy medicinal products (Draft), section 4.9 [16].

The MHRA laboratories at South Mimms produce biological standards including WHO International Standards and NIBSC working reference reagents. They have decades of expertise in biological standardization and can provide help or advice in developing reference materials to support medicinal product and/or diagnostic research and development. The MHRA is, at the time of writing, exploring what reference materials could support phage product development and manufacture. Until these become available, reference reagents and materials could refer to well-characterized bacterial and phage strains available from culture collections that could be used in parallel to test the reproducibility of the DNA extraction and bioinformatics processes for genome assembly and annotation or for validating/providing QC for susceptibility test methods.

### Risk analysis and management

Risk analysis and management come back to quality and working out what processes or other factors pose a risk to it. ICH Q8(R2) [19] is an excellent way to get started on this topic and way of thinking and understand how quality, risk and product development are linked, i.e. identifying risks to the quality of your product, working out where those risks come from and what they look like and then working out how they can be mitigated or controlled. An excellent example is given in EMA guidance for human cell-based medicinal products [20], section 4.1 ‘Risk analysis’, which highlights that risks can be identified through a thorough understanding of the product. Once identified, metrics and methodologies can be used to measure the occurrence and impact of these risks to drive the development of approaches to minimize or mitigate them.

The ICH Q9(R1) guideline for Quality Risk Management [21] defines risk as ‘the combination of the probability of occurrence of harm and the severity of that harm’. The perception of harm may vary for different stakeholders, but for pharmaceuticals, ‘the protection of the patient is of prime importance when managing the risk to product quality and availability, when availability risks arise from quality/manufacturing issues’.

With specific reference to PTPs, risks to product quality imparted by the host strain should be considered, as detailed later on.

### Routine QC testing

Quality control sampling of test batches intended for release ensures that they meet agreed minimal critical quality attributes (CQAs) and product specification(s). Samples should be taken and evaluated at different points along the manufacturing process, especially those points associated with increased risk of an impact on product safety, quality and/or efficacy. The intended outcome of QC sampling and testing is not only to ensure the product is safe but also to help identify where things go wrong – an appropriate sampling strategy will help determine at what point in the manufacturing process the issue arose. More information on Quality by Design can be found in ICH Q8(R2) [19], and a case study for a small molecule drug has been published by the MHRA [22].

### Reference and retention samples

Over and above standard sampling for QC testing for batch release, additional reference and retention samples must be taken as outlined in EudraLex Volume 4 Annex 19 [23]. A **reference sample** is an aliquot (sample) of starting material, packaging material or finished product and should not be used for routine testing; a **retention sample** is a fully packaged unit from a batch of finished product stored for identification purposes.

## Expectations for phage therapeutic products in the UK

Now that general regulatory terminology and thinking have been introduced, expectations for phage therapeutic products will now be discussed in more detail.

### Phage/host cell banks and drug substance

Ideally, PTPs will be traceable to their starting point in a phage bank, and developers should follow general recommendations around documenting the development and maintenance of host cell and phage banks, before moving on to discuss DS identification, characterization and revalidation. It is highly recommended to read published guidance for PTPs; the Ph. Eur. General chapter 5.31 Phage Therapy Medicinal Products [24] and the European Medicines Agency Guideline on quality, safety and efficacy of veterinary medicinal products [25] contain useful information, as does EMA Guideline on quality aspects of phage therapy medicinal products (Draft), section 4.4 [16].

### Banks

ICH has high-level guidance for cell banks [ICH M4Q(R1) [26] section 3.2 .S.2.2], and definitions for Master Cell Bank (MCB) and Working Cell Bank (WCB) can be found in the British Pharmacopeia Appendix XV A. The ICH good manufacturing practice guide for active pharmaceutical ingredients (Q7) [27] contains broad guidance for banking working practices for APIs manufactured by cell culture/fermentation (phage being the latter), especially 18.2 Cell Bank Maintenance and Record Keeping.

All strains should be fully traceable and documented, regardless of their origin (human, xenogeneic or environmental). Where this is not possible, you must demonstrate the absence of adventitious agents and assure the competent authority that there is no risk to recipients of the DS/DP. This can take the form of risk management rather than experimental verification, e.g. avoiding the use of human and/or animal-derived products reduces the risk of acquiring adventitious agents from these sources.

For banks of cells used to express recombinant phage-derived proteins, please consult ICH Q5B Quality of Biotechnological Products: Analysis of the Expression Construct in Cells used for the Production of r-DNA Derived Protein Products [28]. ICH Q5D [29] contains useful information on the use of cells (host bacteria) used to generate both whole phages and phage-derived products.

It is essential to state, define and specify the container(s) used for cell banking, as well as throughout the product’s journey as this may change. Include information on leachables and extractables that may change the composition of the DS. Not all leachables and extractables are a problem, but you need to show that you have investigated them for potential risk to the quality of the product (and patient).

### Quality in phage and host cell banking

The quality of banked material must not only take into consideration the quality of the starting material itself, but also the container system, standard operating procedures and materials used to maintain the bank. In the case of phages, it is worth noting that phages adsorb differently to different surfaces, highlighting the importance of using an appropriate container system for PTPs [30].

### Sterility in banking

In the context of cell-based medicinal products (CBMPs), sterility means free from adventitious organisms, i.e. the absence of any living organism in addition to the active substance (AS). This definition of sterility still applies to microbe-based medicinal products, including phage therapeutics, which can be contaminated with adventitious organisms that present a risk to the patient. It is also important to demonstrate that other materials used in the production and manufacture of the DS and DP are sterile and that the end product is not contaminated with anything that results in a risk to the patient. Production processes should be optimized to minimize or remove adventitious agents and toxic or other impurities; consultation of pharmacopeial methods is recommended. Alternative Methods for Control of Microbiological Quality describes a large number of methods that can be used to determine microbial contamination with Appendix XVI B. Microbiological Examination of Non-sterile Products providing further guidance on tests for detection of [unexpected] pathogenic bacteria. Also see EMA guideline on virus safety evaluation of biotechnological investigational medicinal products [20].

### GMP in cell banking

It is important to apply good manufacturing practice (GMP) as early as possible – including in the maintenance of your phage and host cell banks – and that all materials used to grow, manufacture or store the DS are fully traceable and characterized. A lot of guidance relating to GMP in cell bank establishment and maintenance has been written for mammalian-derived eukaryotic cells, and this is still broadly applicable to bacteriophage and bacterial host-strain banks. EudraLex Volume 4 Guidance on Good Manufacturing Practice for Advanced Therapy Medicinal Products [31] ‘Section 8 Seed lot and cell bank system’ is a useful source and also contains a subsection on ‘Cell stocks/banks and viral seed stocks established outside of GMP conditions prior to the entry into force of Regulation 1394/2007’. See also subsection 7.35 of ICH Q7 [27]. Vaccines, whole cells, whole blood and plasma, blood and plasma derivatives (plasma fractionation) and gene therapy active pharmaceutical ingredients (APIs) are all out of scope of this document, but there is, as always, useful information throughout. Of particular note is Table 1, adapted from ICH Q7 Good Manufacturing Practice for Active Pharmaceutical Ingredients, which highlights where GMP for APIs should start for different manufacturing processes (e.g. fermentation, extraction and purification).

**Table 1.** Summary of GMP application for APIs

Type of manufacturing	Stage at which GMP for APIs should start for different manufacturing processes according to Good Manufacturing Practice for Active Pharmaceutical Ingredients				
Chemical manufacturing	Production of the API starting material	Introduction of the API starting material into process*	Production of intermediate(s)*	Isolation and purification*	Physical processing and packaging*
API derived from animal sources	Collection of organ, fluid or tissue	Cutting, mixing and/or initial processing	Introduction of the API starting material into process*	Isolation and purification*	Physical processing and packaging*
API extracted from plant sources	Collection of plants	Cutting and initial extraction(s)	Introduction of the API starting material into process*	Isolation and purification*	Physical processing and packaging*
Herbal extracts used as API	Collection of plants	Cutting and initial extraction(s)		Further extraction*	Physical processing and packaging*
API consisting of comminuted or powdered herbs	Collection of plants and/or cultivation and harvesting	Cutting/comminuting			Physical processing and packaging*
**Biotechnology: fermentation/cell culture**	**Establishment of an MCB and WCB**	Maintenance of WCB*	Cell culture and/or fermentation*	Isolation and purification*	Physical processing and packaging*
**‘Classical’ fermentation to produce an API**	**Establishment of cell bank**	**Maintenance of the cell bank**	Introduction of the cells into fermentation*	Isolation and purification*	Physical processing and packaging*

Adapted from ICH Q7 Good Manufacturing Practice for Active Pharmaceutical Ingredients, Table 1. The level of GMP stringency increases from left to right; ICH Q7 guidance applies to all boxes containing an asterisk (*). The last two rows of the table (bolded) are most likely applicable to phage therapeutic products. Consult ICH Q7 for more information.

### Strain requalification

Strains held in cell banks used for MAAs are expected to be re-qualified regularly (typically annually for WCB and 3–5 years for MCB) using an agreed set of tests from the original characterization. MCBs should ideally be subject to full characterization with a much smaller set of analytical methods for the WCB.

## Drug substance

### General notes

Again, Ph. Eur. General chapter 5.31 Phage Therapy Active Substances [24] and Medicinal Products for Human and Veterinary Use is a useful source of information [25]. In addition, ICH 5QD [29] General Principles of Characterisation and Testing of Cell Banks (for mammalian cell-derived products and microbially expressed biotherapeutics) gives a good overview of what is needed for the identification and characterization of the DS and why. As stated in the guidance, tests should be carried out to ‘…confirm the identity, purity, and suitability of the cell substrate for manufacturing use’. ‘The extent of characterisation of a cell substrate may influence the type or level of routine testing needed at later stages of manufacturing’. This second sentence also relates to the fact that you need to do a full and comprehensive characterization of the phage/DS. Again, refer to ICH Q8(R2) [19] for context.

Q5D [29] (page 6) also states, ‘Manufacturers are also encouraged to employ “state-of-the-art” methods and technological improvements in cell substrate characterisation and testing as they become available, as long as the specificity, sensitivity, and precision of the newer methods are at least equivalent to those of existing methods’. This is echoed in guidance from the US FDA Process Analytical Technology (PAT) guidance for industry [32], a ‘framework for innovative pharmaceutical development, manufacturing, and quality assurance’.

### Quality

Regulatory guidance pertinent to quality considerations for PTPs DS and DP is outlined in MHRA Regulatory considerations for therapeutic use of bacteriophages in the UK Consideration [1]. The ICH website contains pharmacopeial methods for a number of tests relating to quality, some of which contain specific regional considerations for the EU, USA, Canada and Japan. Again, useful information is contained in the EMA Guideline on quality aspects of phage therapy medicinal products (Draft).

### Quality – identity

ICH Q2(R1) [33] provides guidance on the validation of analytical procedures used to determine ID, impurities and potency, i.e. expectations from competent authorities around accuracy, reproducibility and range. With regard to ID, Q2 states that ‘Identification tests are intended to ensure the identity of an analyte in a sample. This is normally achieved by comparison of a property of the sample (e.g., spectrum, chromatographic behaviour, chemical reactivity, etc) to that of a reference standard’. ICH Q2(R2) [33] provides updated information on ‘analytical use of spectroscopic or spectrometry data’. See also Q14 [34].

One methodological approach for phage and host ID is whole-genome sequencing with full annotation. This should be carried out across the product lifecycle, from banking to batch release. Sequencing quality should be defined, explained and justified, and reference materials, where available, should be used to standardize wet (DNA extraction) and dry laboratory (bioinformatics pipelines) procedures. Regulators would like applicants to state and justify their chosen sequencing quality (e.g. coverage and orthogonal methods), rather than dictating it.

Phages acquire genetic changes over time; applicants should state the expected genetic variation of the DS, e.g. within batches and acquisition of SNPs over a given time frame, and demonstrate that it is not exceeded. Substantial changes may indicate stability problems and/or non-ideal storage conditions, which should be investigated. Each phage is different; product developers are expected to know their product, its characteristics and how to measure them.

For PTPs, the strain (or strains for consortia/cocktail products) or phage-derived material (e.g. lysins or other enzymes) is the AS. For phage-derived products comprised of material other than ‘whole’ phages, an appropriate method should be selected to characterize the DS, e.g. mass spectrometry and flow cytometry; sponsors and manufacturers are best placed to make this decision.

It is not for the regulator to suggest or dictate what method developers/manufacturers should use as this can be restrictive and impede innovation. Many technologies used in this field are in their infancy and will be evolving rapidly; many will become obsolete. Consider what data/evidence is needed, choose an appropriate method and describe it, explain the rationale, highlight the strengths and weaknesses and discuss how these have been accounted for.

### Quality – purity, impurities and contaminants

ICH Q2(R1) [33] provides guidance around the most important assay characteristics for impurity testing assays. ICH Q5C [29] also provides for guidance on purity testing for biologics in the context of stability testing (section 5.3, page 5), which provides useful information. The ICH Q6A Decision Tree document [35] gives a good overview of how to set acceptance criteria for impurities. Note in this document that ‘drug polymorphism’ relates to the phenomenon that DSs can exist in different crystalline forms with different physical properties and is not a reference to the biological definition of polymorphism.

Prophages arising from host bacteria could impart risks to phage products and/or production, e.g. the presence of prophage genes associated with antibiotic resistance or virulence, and/or propensity of the prophage to facilitate generalized or specialized transduction.

Where possible, the use of host strains lacking prophages is preferred, but where this is not possible, prophages should be identified; traced; evaluated for their risk to quality, safety and efficacy; and mitigated where possible.

### Quality – potency

The biological activity of bacteriophages can be determined using a number of well-established, albeit poorly standardized tests. Examples include plaque assays and spot tests. Sponsors should rationalize their methodology choice and, due to the lack of consistency between methodologies, use reference materials wherever possible to provide confidence to the competent authority that every effort has been made to standardize them. We refer you to ICH Q2(R2) [33] and Q2(R2)/Q14. See also ICH Q5C [34] for reference to potency in the context of stability testing.

### Quality – stability

Products are subject to changes in environmental conditions during storage, shipping and handling. The effects of these changes on the stability of your product must be evaluated. When deciding on a formulation strategy for product storage, it is important to consider the intended use or intended user – if your target population lives in a hot and/or humid environment with an unreliable cold chain, you may need to engineer your DS and/or formulate your DP accordingly. ICH Q1 [14] documents in conjunction with Q5C [36], which specifically addresses stability for biological products, should be consulted.

Accelerated degradation studies are recommended where possible, but where it is not, real-world storage data could be used with the shelf life amended as appropriate. Formal stability testing should be done on three batches. Stability testing should focus on where environmental changes or handling could affect quality, safety and efficacy (activity). Take a rational, risk-based approach – if a product will sit in a clear vial exposed to light, photostability might be a consideration for testing, but if the product will not sit in a clear vial exposed to light, then it could be reasonably argued that it is not necessary.

ICH Q2 Analytical Validation [33] provides useful guidance on assay development and suitability for stability (as well as other quality attribute) testing.

## Drug product

The FDA describes a DP as ‘the finished dosage form of the product’ [37]. In the context of PTPs, a DP could comprise the DS (phage) plus excipients formulated into the dosage form (e.g. a capsule). DP is used for the majority of the preclinical work, but DS can be used in some cases, e.g. investigating mechanism/mode of action (MoA), provided that a clear rational explanation for this choice is given. Efficacy should be evaluated with the DP to determine any effects relating to excipients, processing and potential leachables and extractables originating from the container.

Again, it is essential to assess and assure the quality, stability, ‘sterility’ (bioburden) and leachables and extractables of the DP and to define CQAs for the product.

ICH Q8(R2) [19] on pharmaceutical development should be consulted to understand what happens as a product moves along the development pathway and what is expected with regard to characterizing DS versus developing and characterizing DP. There are additional quality requirements for the DP such as pyrogen levels and limits and appearance.

### Quality attributes

A summary of quality attributes for veterinary phage medicinal products was published by the EMA [25] (pages 9–10) in 2023, but the recently published EMA Guideline on quality aspects of phage therapy medicinal products (Draft) [16] provides detailed information on quality expectations for regulatory evaluation of PTMs destined for human use. Again, it will be important to confirm API ID, purities, impurities and contaminants following the guidance highlighted above and in the MHRA document.

ICH Topic Q5C Stability Testing of Biotechnological/Biological Products [36], section 5.3 Purity and Molecular Characterisation states, ‘**purity is a relative term**. Due to the effect of glycosylation, deamidation or other heterogeneities, **the absolute purity of a biotechnological/biological product is extremely difficult to determine.** Thus, the purity of a biotechnological/biological product should be typically assessed by more than one method’. Regulators know that biological products are highly complex and vary from batch to batch – this is not a problem if you understand and define the variability and show that it is within acceptable limits. Reference standards are very helpful with regard to the demonstration of purity and should be used where possible.

Impurities can be process- or product-related, and the presence of an impurity or contaminant is not necessarily a problem if it is known, characterized and either mitigated or shown not to be a risk to the patient; a risk-based approach should be used with regard to impurities and contaminants.

Fermentation is used widely for the production of biologics and contaminants, and the relevant assays are, by and large, well defined. Examples include host cell protein (HCP), host cell DNA and pyrogens. As well as the Ph Eur General chapter 5.31 Phage Therapy Active Substances and Medicinal Products for Human and Veterinary Use on host cell protein (HCP) [25], additional EMA guidance can provide some help on this topic [38,39] [see ‘Host cell proteins testing, drug substance specification (3.2.S.3.2, 3.2.S.4.1, 3.2.S.4.5)’], while WHO Guidelines for the production and QC of monoclonal antibodies and related products intended for medicinal use [40] contain some brief guidance in sections A.5.3.2 and A.5.6.8.1 for HCP tests and limits, as well as tolerances for residual host cell DNA (A.5.3.3). See also ICH 6QB [18] section 2.1.4 Impurities along with Q6B Appendix section 6.2.1 Process-related impurities and contaminants. Both the United States Pharmacopeia (USP) and the European Directorate for the Quality of Medicines and Healthcare have general chapters on HSP and residual host cell DNA, and the USP has physical standards for residual DNA testing of both Chinese hamster ovary cell and *E. coli*-derived biotherapeutic products.

#### Pyrogen testing

It is important to quantify pyrogen levels in your product, either because they could be a contaminant that causes adverse events, or because pyrogens are part of the DP and necessary for the desired physiological response in the recipient.

The expected levels and types of pyrogen(s) will be determined using a risk assessment that considers all manufacturing steps, raw materials, equipment and the biology of the production system, and this will dictate which assay is most appropriate to measure it. There are bacterial endotoxin tests (BETs) and pan-pyrogen tests:

- BET, including the LAL and its recombinant variants, only detects endotoxins (Bacterial Endotoxins, Ph. Eur. method 2.6.14).
- MAT (Monocyte Activation Test) detects all pyrogens (see BP Appendix XIV H. Monocyte-Activation Test/Ph. Eur. 2.6.30). This guidance includes information on how to interpret the test results, as well as a guide to calculate contaminant limit concentration.
- Rabbit Pyrogen Test detects all pyrogens but is being phased out (by 2026 for Ph. Eur.) due to the availability of alternatives (MAT).

Where pyrogen(s) is an expected contaminant (i.e. not a part of the DP and required for efficacy), there is an upper limit specification (related to administration route). In such cases, a statement stating that the batch does not need to exceed the limit (e.g. <0.5 IU mg^−1^) is sufficient. It is the responsibility of the manufacturer to do the risk assessment and present data and findings to the regulator.

### Quality – biological activity/efficacy

When measuring the biological activity of the DP, it is usually **efficacy** that is being assessed, rather than **activity**. Bioassays will be needed that measure the bioactivity/potency of the DP and that it is able to exert the desired effects at the desired site. Measuring biological activity is needed to demonstrate that DP batches can be reproducibly and consistently produced and meet defined CQAs. Potency assays should be validated prior to clinical trials, and we recommend additional potency tests, linked to the MoA of the product, where possible. This is of the utmost importance, especially when evaluating non-living cell products, e.g. non-viable micro-organisms, microparticles or lysates.

### Stability

Changes to the dosage form (same AS but different pharmaceutical product type) will require new, albeit possibly reduced, stability data for the new formulation. Refer to ICH Q1C [14].

### Appearance

Visual inspection of DP is a common part of batch release (medicines control). It is a simple way to tell if there is something wrong with a batch, e.g. contamination, manufacturing issues or counterfeit products. Visual inspection should be of the container and content. Assessment could include colour, opacity, flocculation or the presence of particulate that should not be there.

### Container and closure system

Containers/closure systems must be fully documented and characterized as stated elsewhere in this document. Furthermore, as stated in ICH Q1A(R2) [14] section 2.1.4, page 2, ‘stability studies should be conducted on the drug substance packaged in a container closure system that is the same as or simulates the packaging proposed for storage and distribution’.

### Changes in composition

If changes are made to the product’s DS after approval of a licensing application, a variation needs to be submitted to the competent authority (MHRA in this case). Advice on post-MA variations can be found on the EMA Post-authorisation page [41]. Note that Type IA variations are those that have no impact on product quality, safety or efficacy; a change in the taxonomy of a strain would likely count as a Type IA variation, but guidance can change, so it is advisable to check the advice if/when a change is required. Further information on MA variations is available on the MHRA website; not also section 6.1 in the EMA Guideline on quality aspects of phage therapy medicinal products (Draft) [16].

At the time of writing, the MHRA has not received any applications for PTPs. With regard to ‘cocktail’ or mixed phage products, whose composition will change over time to ensure continued efficacy, it is envisaged that the initial marketing authorization will be for a defined cocktail of phages. If the marketing authorization holder wishes to change any of the phages, they would submit a variation to the marketing authorization proposing the new cocktail. The variation submission would contain a data package justifying the change without recourse to further clinical studies. If assessed as inadequate, the MHRA would ask for a clinical study with the new phage cocktail, but this will partly depend on what was qualified by the clinical trials and how different the new phage is from the current phage it’s replacing.

### Manufacturing

DP will ideally be used for the majority of the preclinical work, as well as *all* the clinical work, which is relevant to cocktail/mixed phage products, but not necessarily single use/compassionate use/experimental use. We have already stated that cell banking should be carried out under GMP, so you should by now be familiar with quality systems and applying a risk-based approach.

It is worth noting that preclinical and clinical trials are partly evaluating the manufacturing process; they determine whether the process (including packaging) generates a DP that is safe and can be used safely in the clinical setting. Further information and guidance on this can be read in Volume 4 EU Guidelines to Good Manufacturing Practice Medicinal Products for Human and Veterinary Use Annex 13 Investigational Medicinal Products [42] and Detailed Commission guidelines on good manufacturing practice for investigational medicinal products for human use, pursuant to the second subparagraph of Article 63(1) of Regulation (EU) No 536/2014 [43]. ATMPs are out of scope of this guidance, and specific documentation pertaining to GMP for ATMPs can be found in EudraLex The Rules Governing Medicinal Products in the European Union Volume 4 Good Manufacturing Practice Guidelines on Good Manufacturing Practice specific to Advanced Therapy Medicinal Products [31].

## Good manufacturing practice

### What is GMP?

GMP ensures that products are of appropriate and defined quality, produced consistently, fit for purpose and ‘meet the requirements of the market authorisation or product specification’. GMP sits alongside GLP and good distribution practice (GDP) to ensure products obtained from the licensed supply chain are stored, transported and handled in accordance with the MA or product specification so that patients receive products that are as intended. GMP and GDP licensure and inspections in the UK are overseen by the MHRA Inspectorate. There are different manufacturing licences depending on your requirements; the MHRA publication lists all the regulatory requirements for GMP production in relation to PTPs, and information on how to apply for a licence to manufacture in the UK is detailed on the MHRA website [44]. Companies applying for a marketing authorization (also known as a product licence) need to have a manufacturer licence first which will be granted providing the product is in the process of being approved. Up to this point, the drug will have been manufactured under an investigational medicinal product (IMP). As with all manufacturing licences, you will be subject to inspections to ensure compliance. The MHRA Inspectorate official blog has a lot of useful information, as well as FAQs pertaining to GMP, and a summary of pertinent licences is summarized in Table 2.

**Table 2.** Types of licences manufacturers of DPs must have, and what each type of licence covers

Type of licence	Purpose
Manufacturer’s/importer’s licence (MIA)	Manufacture and/or assemble licensed medicinal products, including export to a country outside the EEA, or (**b**) import licensed medicinal products from countries outside the EEA
Manufacturer’s licence for IMPs [MIA(IMP)]	Manufacture IMPs for use in clinical trials
Manufacturer’s ‘specials’ licence (MS)	Manufacture of unlicensed medicines ‘specials’, or (**b**) importation of unlicensed medicinal products from outside the EEA

The MHRA publishes the Orange Guide (Rules and Guidance for Pharmaceutical Manufacturers and Distributors) [45], which contains information and legislation relating to the manufacture and distribution of human medicines, and serves as a single source of European and UK guidance and UK legislation on the manufacture and distribution of human medicines, ASs and brokering medicines. The Orange Guide sits behind a paywall, so if this is not accessible, EU EurdaLex – Volume 4 – GMP Guidelines [46] is a good source of information and provides extensive and detailed information on everything from facility and personnel requirements to documentation and IT infrastructure. Annex 2 helps interpret the principles and guidelines of GMP for *biological* medicinal products. This annex contains revisions for biological ASs and is applicable to natural and GMM PTPs where the modification is NOT related to MoA. In addition, ICH Q7 [27] guidance for GMP applies to biologicals, with section 18 specifically relevant to products produced by fermentation, i.e. phages and phage-derived proteins. Phages fitting the gene therapy classification are out of scope of ICH Q7. Where PTPs are ATMPs, they should be developed in accordance with Part IV GMP for ATMPs.

#### Continuous manufacturing

ICH Q13 [47] guideline on Continuous Manufacture of Drug Substances and Drug Products provides scientific and regulatory information that may be of use for products produced through continuous manufacture.

### GMP QC

To facilitate product release and ensure patient safety, assays/product evaluation must be carried out during the manufacturing process, as well as on the final product to verify that the product meets CQAs. PAT guidance for CMC [48] contains non-mandatory guidance on how to evaluate product quality along its development and manufacturing journey to provide confidence to the regulator that the product meets quality requirements. There is a lot of useful guidance in this document, and it again highlights that by taking responsibility and being proactive, time and money (and disappointment) can be saved.

#### In-process controls and DP release criteria

The DP should be tested at points along the processing/manufacturing pipeline, the extent of which will depend on the level of risk identified during risk assessment of product development and manufacturing tests and refinement. ‘Open’ processing may, for example, warrant more sterility testing than closed processing. Use a risk-based approach to determine when and where in the processes that tests would be appropriate.

Types of in-process measurement (as defined in FDA Process Analytical Technology [48]) include the following:

1. At-line: measurement where the sample is removed, isolated from and analysed near the process stream.
2. On-line: measurement where the sample is diverted from the manufacturing process and may be returned to the process stream.
3. In-line: measurement where the sample is not removed from the process stream and can be invasive or non-invasive.

Section 18.16 in the Q7 ICH GMP guide for APIs [27] lists several process controls that should validate products and processes. Again, where PTPs are produced via continuous manufacturing, you should consult ICH Q13 Continuous Manufacturing of Drug Substances and Drug Products [47].

### Changes in manufacturing

It is important to demonstrate batch-to-batch consistency. Related documentation can be found in section 4.2.8 Comparability in the EMA CBMP recommendations, the ICH Q5E Comparability of Biotechnological/Biological Products [49] and the EMA Q and A on Comparability considerations for ATMPs [50].

Scale can change dramatically from the engineering runs used to set up the GMP process through to the commercial process. For example, a monoclonal antibody DP can go from a 50 l production bioreactor to 10,000 l during preclinical to phase III studies. We recommend that the commercial process (full scale) is established before the pivotal clinical trials so that it is 'clinically qualified' for the MAA. The clinical qualification of a manufacturing process is very important to regulators to demonstrate quality, safety and efficacy of the DP.

### Labelling

As highlighted in the Phar Eu general chapter [24], labelling should comply with local requirements.

## Preclinical characterization

Up to the point of preclinical characterization, quality has been the main driver, and sufficient evidence must be provided to the regulator to give them confidence that the product is of high enough quality to be developed as a drug. To put a drug into humans, there must be sufficient evidence of safety. Note that CTD documentation for Module 4 (non-clinical study reports) should be structured according to ICH M4S [51].

### General considerations

Helpful guidance for preclinical work can be found in the ICH CTD chapter on safety [M4S(R2)] [51] and the ICH guideline S6 (R1) [52] (addendum published in 2011 provides updated information). Preclinical safety studies are needed prior to initiating first-in-human studies, but also throughout clinical development. The preclinical programme should be designed considering the intended use/end user.

Understanding and characterizing biological activity is necessary to define the MOA, i.e. what does the product do – at a high level – to ameliorate disease/patient health status? This must be understood as it highlights any risk the product could pose to a recipient/patient and what risk management will be put in place to mitigate that risk.

S6 (R1) [52] (page 3) also points out that preclinical studies should be done under GLP but acknowledges that this is not always possible, and that in such cases, ‘Areas of non-compliance should be identified and their significance evaluated relative to the overall safety assessment’, i.e. where GLP is not possible, it should be demonstrated that there is no significant impact on safety.

For PTPs fitting the ATMP classification, see also ICH S12 Nonclinical Biodistribution Studies for Gene Therapy Products [53] and the EMA Guideline on the non-clinical studies required before first clinical use of gene therapy medicinal products (EMEA/CHMP/GTWP/125459/2006) [54]. As is the case with other GTMP guidance, much of the data required is covered under the biologics guidance, and much of the GTMP-specific requirements is non-pertinent due to the nature of phages; it is highly recommended to speak to the competent authority to determine the level of work needed to demonstrate safety and efficacy of your PTP.

Finally, ICH S11 Nonclinical Safety Testing in Support of Development of Paediatric Pharmaceuticals [53] should be consulted if the PTP is intended to be used in children below the age of 12. The nature of phages means that it is unlikely that a significant amount of non-clinical work will be needed, but this document explains how this is determined and what to do.

### Designing a preclinical work package

The preclinical work package may comprise *in silico*, *in vitro*, ex vivo and, where appropriate, *in vivo* data to demonstrate suitability for the intended use, efficacy and safety. Preclinical work should focus on characterizing and documenting the biological/biochemical properties of the product to show it is safe, provide data to instruct subsequent clinical trials and provide a high-level MoA.

You will need to demonstrate that DP batches can be reproducibly and consistently manufactured and meet defined CQAs. Bioassays will be needed to measure the bioactivity/potency of the DP, and experimental approaches that demonstrate that the DP is viable and able to exert the desired effects at the desired site are required. The MHRA neither supports, rejects nor indicates any specific model and encourages the sponsor/manufacturer to choose the most suitable and appropriate one with a clear explanation for that choice. Furthermore, using animal models that do not add value to the evidence base used to evaluate medicine quality, safety and efficacy is discouraged.

For PTPs targeting microbiome communities, Fekete *et al*. provide an excellent summary of functional assays, including microbiome-immune system models that can be used in lieu of animal models [55].

### Pharmacology, biodistribution and toxicology

Regulatory challenges around biotherapeutics and ATMPs have paved the way for case-by-case regulatory discussions and the acknowledgement that there is ‘no one size fits all’ solution. In the subsections below, we have referenced existing guidance that can help you develop a dossier, but if ever in doubt, the regulator evaluating the MAA should be consulted.

#### Biological activity/pharmacodynamics

To help identify what metrics and markers demonstrate how a product is behaving *in vivo*, the following questions could be asked:

- What measurable characteristics (metrics) are associated with the disease of interest?
- Which metrics/disease characteristics does the PTP rectify/alter?
- How can changes in disease characteristics reflecting efficacy (or not) be measured before and after treatment?
- What disease characteristics could indicate a worsening of the patient’s condition, or an adverse drug reaction?
- What disease characteristics could present a risk to the patient when exposed to the PTP, e.g. loss of barrier function related to disease pathology that might present a risk of translocation (i.e. toxicity)?
- What metrics can be used to monitor patient health?
- What preparations/risk mitigation can be put in place in the event of an adverse drug reaction?

PTPs have some interesting properties that may make traditional ADME (absorption, distribution, metabolism and excretion) studies challenging. This is not unique to PTPs, however, and regulators are experienced in reviewing products that sit outside of convention; if traditional ADME studies are not suitable, explain why and provide an alternative.

### Toxicity testing and animal models

PTPs have been used for over a century, and there is substantial evidence of their safety, but safety doesn’t relate only to the API; it also concerns excipients, the container system and any other components. Again, use a logical risk-based approach to determine where there may be risks to product quality and, therefore, the patient, and use this to develop your safety testing programme.

ICH S6 (R1) [52] and CHMP guidance on human CBMPs [20] section 4.3.2. are useful information sources; the latter states, ‘The need for toxicological studies depends on the product [and where] conventional study designs may not be appropriate, the scientific justification for the models used, or the omission of studies, shall be provided’.

Also note sections 2.1 and 2.2 of Part II: Addendum for ICH S6 (R1) [52]: ‘For products that are directed at a foreign target such as bacteria and viruses, in general no reproductive toxicity studies would be expected’.’ If in doubt, scientific advice can be sought from the MHRA.

Be aware that toxicity may not be evident at first and may become apparent over the lifetime of a product due to, for example, changes in manufacturing materials or processes, long-term accumulation or persistence of DP components (perhaps arising from poor clearance in patients or the elderly) or use with other medications, including newly licensed drugs. This should be monitored as part of ongoing clinical studies and post-marketing authorization pharmacovigilance.

### Administration/dose escalation

Administration and frequency of dose in animal studies should match proposed clinical use as closely as possible. Again, regulators are used to seeing applications where dosing levels are not based on traditional preclinical pharmacology and toxicology experiments, and advice can be sought from the regulator if in doubt. With specific regard to phages, the relationship between phage dose (MOI) and favourable outcomes is not yet clear [56]. Dosing should be based on scientific evidence (from the preclinical work package including scientific literature where appropriate) and clearly explained in the clinical trial application.

### Immunogenicity

Immunogenicity is a major concern for biotherapeutics as they can induce the formation of anti-drug antibodies that neutralize their biological activity and/or cause severe adverse immunological reactions in recipients/patients. Bacteriophages can be immunogenic; the recent retrospective, observational analysis of the first 100 consecutive BT cases of difficult-to-treat infections from the Belgian consortium reported neutralizing antidrug antibodies in 38.5% of cases [56]. If anti-phage antibodies neutralize or promote clearance of the phage, they may reduce efficacy and limit repeated use. The presence of aggregated material in biotherapeutic products can have serious adverse effects *in vivo* [57,58]. Sponsors should evaluate the immunogenicity of products using the appropriate models as part of the preclinical work package and monitor immunogenicity in human studies.

### Toxicology

Naturally occurring bacteriophages are highly abundant in the biosphere and do not target mammalian cells. It is unlikely that bacteriophage will elicit toxicity in humans and animals, but intravenous administration of manufactured and/or engineered PTPs could present a risk of exposure to pyrogens or other manufacturing artefacts, or unknown risks imparted through genetic engineering, e.g. novel epitopes or other changes that could cause harm to the recipient.

ICH S6 (R1) [52] (and other guidance documents, e.g. CHMP guidance on human CBMPs) conveys the message that genotoxicity and carcinogenicity for biotherapeutics are low and should only be investigated if there is a perceived risk based on MoA.

### Antibiotic cooperativity and antagonism

It is known that both cooperativity and antagonism are seen with phage-antibiotic combinations [56]. It will likely be part of any development programme to determine the efficacy of phage products against various antibiotic backgrounds for both efficacy and safety reasons. It is recommended that reasonable efforts are made to characterize phage/antibiotic interactions corresponding to the indication you are seeking to treat. One should also be mindful that not all national and international recommendations pertaining to standard or care antibiotic use are the same, so be mindful of healthcare/HTA guidelines in the areas the PTP is destined for.

## Clinical trials

### General overview of clinical data for MA applications

CTD Module 5 clinical study reports should be presented in the order outlined in Guideline M4E, specifically, ICH E3 Structure and Content of Clinical Study Reports (CPMP/ICH/137/95) [59]. In a submitted dossier, a clinical overview (2.5) and a clinical summary (2.7) are provided in Module 2 (summary reports) with the data supporting those summaries in Module 5: clinical study reports. ICH guidance on efficacy can be found in M4E(R2) [60], which covers the efficacy information you need to provide in Module 2 **and** Module 5. Additional information and training material pertaining to the M4E(R2) document is available from the ICH website.

The Clinical Summary should contain an analysis of pharmacokinetics (PK) and pharmacodynamics (PD) data; where traditional PK/PD studies are not appropriate, surrogate metrics and methodologies should be used to describe and measure DP characteristics. Applicants should provide data necessary to demonstrate safety and efficacy; if in doubt, speak to the regulator and get advice (be sure to document any discussions/advice given in the dossier).

### Clinical trials for PTPs

Despite their long history of use in Georgia, Russia and Poland, there is a lack of clinical trial data in the form more commonly submitted for medicinal products; at the time of writing, no clinical trials for licensed PTPs have been conducted in the UK, and we strongly recommend speaking with the MHRA about the development of your clinical programme. It is important to remember that not all regulators – or the healthcare ecosystems they sit in – are the same and that this can affect regulatory expectations about medicinal products. For example, in the UK, the decision about whether to pay for a medicine for use in the National Health System (NHS) is a decision for the HTA boards, not the medicine regulator, and, therefore, the primary concern of the regulator is patient safety and evaluating evidence of claims made by the sponsor; it is the HTAs that are concerned with efficacy and whether the cost is outweighed by the benefit. There is more information about HTA in the section below. MHRA Guidance on Clinical trials for medicines: apply for authorisation in the UK [61] provides information on how to apply for a clinical trial including eligibility, phases, model IMPDs and costs, and how to make changes to your application provides general information around MHRA clinical trial authorizations. To check the status of a clinical trial submission, or if you have a general clinical trial or technical query regarding an application you have submitted or plan to make, call our helpline (weekdays, 8 : 30 am to 4 : 30 pm) on 020 3080 6456 or contact the team through clintrialhelpline@mhra.gov.uk.

General ICH guidance for clinical trials can be found in the ICH Efficacy Guidelines documents – not all guidance documents are applicable as they relate to patient groups and study types outside of that for which PTPs are intended; consult the relevant information as required. Clinical trial guidance for GTMPs is contained within EMA Guideline on the quality, non-clinical and clinical aspects of gene therapy medicinal products (EMA/CAT/80183/2014) [62].

Products submitted for marketing authorization application (MAA) to the MHRA should be entered into UK PharmaScan, a database of information about new medicines, indications and formulations that are ~3 years away from UK availability, or at the start of a phase 3 clinical trial and remain on the system until available in the UK. Horizon scanning organizations use the data for the following:

- pathway and system planning
- identification of transformative medicines for the Accelerated Access Collaborative
- development of HTA schedules by NICE, SMC and AWMSG
- production of briefings and resources for the NHS in England, Scotland and Wales
- local planning such as formulary development, service design and budgeting
- informing national planning and prioritizing engagement with companies

### PK and PD

It is important to show that products get to where they are needed and don’t cause off-target effects that present a risk to the recipient. Despite a century of use, there is little information pertaining to PK studies, yet some information in the scientific literature on PTPs in clinical use may be of value [30]. Some key points highlighted in this review include the following:

- The choice of administration route is likely to have a major impact on phage PK
- Body fluids can impact phage lytic activity
- Phage PK is complicated by the unique ability of phages to amplify themselves at the site of infection (auto-dosing) wherein phage counts far exceed the exogenously administered dose.

Despite these challenges, some PK metrics can and should be measured, for example, bioavailability, clearance (especially for products targeted to the bladder or gut), maximal concentration (may be relevant for systemic infections), half-life and distribution. Some of these metrics may be related to MoA and should be explored.

With regard to PD, primary PD relates to on-target effects (safety and efficacy), while secondary PD relates to off-target effects. Again, use a risk-based approach in relation to the quality and biological activity of your product to investigate PD as appropriate. Again, interesting information is discussed in the 2022 review [30] that may be helpful in designing your PD studies.

### Inclusion of women of childbearing potential

See ICH S6 (R1) [52] section 5.4. Exclusion of women of childbearing age or inclusion criteria necessitating the use of contraceptives in clinical trials for PTPs will probably be required if animal data demonstrating safety is lacking. In such cases, if the product is given once in a trial, then a pregnancy test prior to that administration can be readily applied for all relevant females, who only continue with the trial if the test is negative. If the DP is given more than once, then repeated pregnancy testing should be carried out with a positive test being a withdrawal from the trial. Such criteria would be likely to be suitable, along with contraceptive requirements in the protocol. As stated in CHMP guidance for human CBMPs section 4.3.2 (Toxicology): ‘The need for reproductive studies is dependent on the [product] and should be considered on a case-by-case basis’ [63].

## Phase IV/pharmacovigilance

It is a requirement that outcomes from the use of a product are monitored to identify long-term effects not detected in clinical trials; section 4.4.7 Pharmacovigilance and Risk Management Plan of the CHMP Guideline in Human CBMPs [20] is broadly applicable. There is guidance on pharmacovigilance in the UK and the EU – note that the Northern Ireland market sits inside the EU framework while Great Britain does not. Consult MHRA guidance on pharmacovigilance for the UK and the EMA Pharmacovigilance: post-authorization for EU guidance.

Transfer of PTPs to healthy individuals and the environment is something that needs to be considered and potentially controlled. Evidence of how the transfer of PTPs beyond the patient has been controlled for should be provided where appropriate. Guidance on pharmacovigilance can also be found in ICH documents E2A-2EF [64].

### Environmental impact

As stated above, the submission of an ERA as part of an MAA is required for pharmaceutical GMMs; further information on developing an ERA is provided in the CHMP Guidance on environmental risk assessments for medicinal products consisting of, or containing, genetically modified organisms (GMMs) (EMEA/CHMP/BWP/473191/2006 – Corr) [5]. The implementation of blue/green prescribing [65] practices in response to the persistence of prescription drugs in the environment highlights the importance of clear guidance to patients, pharmacists and clinicians on good practice around the use and disposal of GMMs.

Two initiatives – the Antibiotic Manufacturing Standard and a BSI Kitemark^™^ for minimized risk of antimicrobial resistance – have been facilitated by the National Standards Body for the UK and the globally accredited assurance body British Standards Institution (BSI).

These initiatives are defining and driving implementation of responsible antibiotic manufacturing practices to help mitigate the impact of AMR in the environment and allowing manufacturers to evidence conformance to the Antibiotic Manufacturing standard.

The BSI-led development of a Publicly Available Specification standard, which will align medicine manufacturers, regulators, governments, NGOs, academia and healthcare systems on the methodology to calculate environmental impact across the lifecycle of a medicine, takes into account how environmental impact is calculated across the individual medicine product categories.

## HEALTH TECHNOLOGY ASSESSMENT IN THE UK

In the UK, the MHRA grants market access based on the evaluation of the evidence submitted in the MAA. If a licence is granted, HTA bodies will usually make a recommendation to the healthcare system about whether the medicine should be made available based on its value for money to patients, which is estimated through an evaluation of the available evidence.

In Scotland, the SMC provides advice to NHS Scotland about the value for patients of newly licensed medicines. In England, the HTA body is called the NICE; Wales and Northern Ireland tend to adopt the recommendations published by NICE. Note that the SMC does not charge companies to submit for an evaluation, but there is a charge for submitting an application for an evaluation by NICE, although this fee is reduced for small companies.

HTA organizations tend to make recommendations about whether a product should be used in the NHS based on a review of the clinical evidence and cost effectiveness (how well the treatment works in relation to how much it costs the NHS, i.e. does it represent value for money?). The NICE process document on health technology evaluations [66] provides an outline of the NICE process for a technology appraisal. Not all medicines need to be evaluated by an HTA body, and a summary of products that are usually evaluated by NICE is available [67]. Additionally, the remit of NICE is to decide whether medicines can be made available on NHS, so if a drug is not recommended by NICE, a private company could still provide it.

NICE recognizes that antimicrobial resistance is an urgent global challenge that is negatively impacted by a lack of commercial attractiveness due to cost expectations and restricted use. To uncouple volume sold with revenue generated, NICE and NHS England have launched a new approach to incentivize companies to invest in new antimicrobials by evaluating and paying for selected products based on overall value added rather than the number of prescriptions written. This assessment is done in a different way from other medicines, which are normally evaluated via a process called a technology appraisal. Currently, the subscription model [68] encompasses only traditional (small molecule) antimicrobials, but the eligibility criteria may be expanded to include non-traditional (e.g. phage and microbiome) and reviewed regularly.

Familiarization with the types of evidence requirements, and the process for existing antimicrobial evaluation, will likely still be useful. NICE published a manual for its health technology process in 2022; a summary of expectations relating to antimicrobials can be found in Annex 7: health technology assessment process [69] but may change if/when eligibility criteria also change. The approach to antimicrobial evaluation at NICE is different to the established approach NICE takes to evaluate the majority of medicines it sees, but principles of this established technology appraisal approach may be followed, and so awareness of the evidence requirements (linked above) for a technology appraisal may also be useful.

NICE provides a NICE Advice service to support companies seeking to enter the NHS market. This advice can help prepare for a NICE evaluation, or engagement with NHS payers or commissioners. This is a fee-based service.

## Abbreviations

APIs: active pharmaceutical ingredients
AS: active substance
ATMP: advanced therapeutic medicinal product
AWMSG: All Wales Medicines Strategy Group
BETs: bacterial endotoxin tests
BSI: British Standards Institution
CBMPs: cell-based medicinal products
CQAs: critical quality attributes
CTD: Common Technical Document
Defra: Department for Environment, Food and Rural Affairs
DME: absorption, distribution, metabolism and excretion
DP: drug product
DS: drug substance
ERA: environmental risk assessment
FDA: Food and Drug Administration
GDP: good distribution practice
GLP: good laboratory practice
GMM: genetically modified micro-organism
GMP: good manufacturing practice
HCP: host cell protein
HMRs: Human Medicines Regulations 2012
HSE: Health and Safety Executive
HTA: health technology assessment
ICH: International Council for Harmonisation of Technical Requirements for Pharmaceuticals for Human Use
IMP: investigational medicinal product
MA: market authorization
MAA: marketing authorization application
MAT: Monocyte Activation Test
MCB: Master Cell Bank
MoA: mechanism of action
NHS: National Health System
NICE: National Institute for Health and Care Excellence
NRAs: national regulatory authorities
PAT: Process Analytical Technology
PD: pharmacodynamics
PK: pharmacokinetics
PTMs: post-translational modifications
PTPs: phage therapeutic products
QCs: quality controls
SMC: Scottish Medicines Consortium
USP: United States Pharmacopeia
WCB: Working Cell Bank
