# Corrigendum: Interpretation guidance for MHRA regulatory considerations for phage therapeutic products

**DOI:** 10.1099/mic.0.001670

**Published:** 2026-02-24

**Authors:** Carmen Coxon, Elizabeth Bell, Evelien Adriaenssens, Jason Clark, Joe Edwards, Tas Gohir, Francesca Hodges, Josh Jones, Cath Rees, Annette Sansom, Darren Smith, Mark Sutton, Clare Trippett, Dann Turner

**Affiliations:** 1 MHRA Science Campus, Blanche Lane, South Mimms, Potters Bar, Hertfordshire EN6 EQG, UK; 2 National Institute for Health and Care Excellence, Manchester M1 3BN, UK; 3 Innovate UK Phage Innovation Network Advisory Group, Unit 218, Business Design Centre, 52 Upper Street, Islington, London N1 0QH, UK; 4 Bacteriophages and Viromics, Quadram Institute, Rosalind Franklin Road, Norwich Research Park, Norwich NR4 7UQ, UK; 5 NexaBiome, West of Scotland Science Park, Block 2 Kelvin Campus, 2317 Maryhill Road, Glasgow G20 0SP, UK; 6 Aparon, Hill Farm Middleton Lane, Middleton, Tamworth B78 2BN, UK; 7 Guy’s and St Thomas' NHS Foundation Trust, The Grain House, 46 Loman Street, London SE1 0EH, UK; 8 Innovate UK Business Connect, Unit 218, Business Design Centre 52 Upper Street, Islington, London N1 0QH, UK; 9 Antimicrobial Discovery Development and Diagnostics (AD3), UK Health Security Agency, Manor Farm Road, Porton Down, Salisbury SP4 0JG, UK; 10 School of Biosciences, University of Nottingham, Nottingham NG7 2RD, UK; 11 Campden BRI, Station Road, Chipping Campden GL55 6LD, UK; 12 Applied Sciences, Northumbria University, Newcastle upon Tyne NE1 8ST, UK; 13 Institute of Pharmaceutical Sciences, King’s College London, London WC2R 2LS, UK; 14 Centre for Process Innovation, 1 Union Square, Central Park, Darlington DL1 1GL, UK; 15 University of the West of England, Coldharbour Lane, Bristol BS16 1QY, UK; 16 School of Applied Sciences, College of Health, Science and Society, University of the West of England, Coldharbour Lane, Bristol BS16 1QY, UK

The authors wish to add the following disclaimer to their article:

‘This document reflects the views of subject matter experts from the Innovate UK Phage Innovation Network and the MHRA Science Campus and should not be construed to represent the official view of any given regulatory authority.’

In line with this request, the version of record will also be updated to include the disclaimer text.

